# Interaction of alginate with nano-hydroxyapatite-collagen using strontium provides suitable osteogenic platform

**DOI:** 10.1186/s12951-022-01511-9

**Published:** 2022-06-28

**Authors:** Ayla Hassani, Çığır Biray Avci, Sajed Nazif Kerdar, Hassan Amini, Meisam Amini, Mahdi Ahmadi, Shinji Sakai, Bakiye Goker Bagca, Neslihan Pınar Ozates, Reza Rahbarghazi, Ali Baradar Khoshfetrat

**Affiliations:** 1 Chemical Engineering Faculty, Sahand University of Technology, Tabriz, 51335-1996 Iran; 2 Stem Cell and Tissue Engineering Research Laboratory, Sahand University of Technology, Tabriz, 51335-1996 Iran; 3 Department of Medical Biology, Faculty of Medicine, Ege University, Izmir, Turkey; 4 Stem Cell Research Center, Tabriz University of Medical Sciences, Tabriz, Iran; 5 Department of General and Vascular Surgery, Tabriz University of Medical Sciences, Tabriz, Iran; 6 Student Research Committee, Tabriz University of Medical Science, Tabriz, Iran; 7 Drug Applied Research Center, Tabriz University of Medical Sciences, Tabriz, Iran; 8 Division of Chemical Engineering, Department of Materials Science and Engineering, Graduate School of Engineering Science, Osaka University, Osaka, 560-8531 Japan; 9 Department of Applied Cell Sciences, Faculty of Advanced Medical Sciences, Tabriz University of Medical Sciences, Tabriz, Iran

**Keywords:** Alginate-Nanohydroxyapatite-Collagen hydrogel, Ionic cross-linkers, Osteogenic capacity, Cell dynamic growth, Critical-sized bone regeneration

## Abstract

**Background:**

Hydrogels based on organic/inorganic composites have been at the center of attention for the fabrication of engineered bone constructs. The establishment of a straightforward 3D microenvironment is critical to maintaining cell-to-cell interaction and cellular function, leading to appropriate regeneration. Ionic cross-linkers, Ca^2+^, Ba^2+^, and Sr^2+^, were used for the fabrication of Alginate-Nanohydroxyapatite-Collagen (Alg-nHA-Col) microspheres, and osteogenic properties of human osteoblasts were examined in in vitro and in vivo conditions after 21 days.

**Results:**

Physicochemical properties of hydrogels illustrated that microspheres cross-linked with Sr^2+^ had reduced swelling, enhanced stability, and mechanical strength, as compared to the other groups. Human MG-63 osteoblasts inside Sr^2+^ cross-linked microspheres exhibited enhanced viability and osteogenic capacity indicated by mineralization and the increase of relevant proteins related to bone formation. PCR (Polymerase Chain Reaction) array analysis of the Wnt (Wingless-related integration site) signaling pathway revealed that Sr^2+^ cross-linked microspheres appropriately induced various signaling transduction pathways in human osteoblasts leading to osteogenic activity and dynamic growth. Transplantation of Sr^2+^ cross-linked microspheres with rat osteoblasts into cranium with critical size defect in the rat model accelerated bone formation analyzed with micro-CT and histological examination.

**Conclusion:**

Sr^2+^ cross-linked Alg-nHA-Col hydrogel can promote functionality and dynamic growth of osteoblasts.

**Graphical Abstract:**

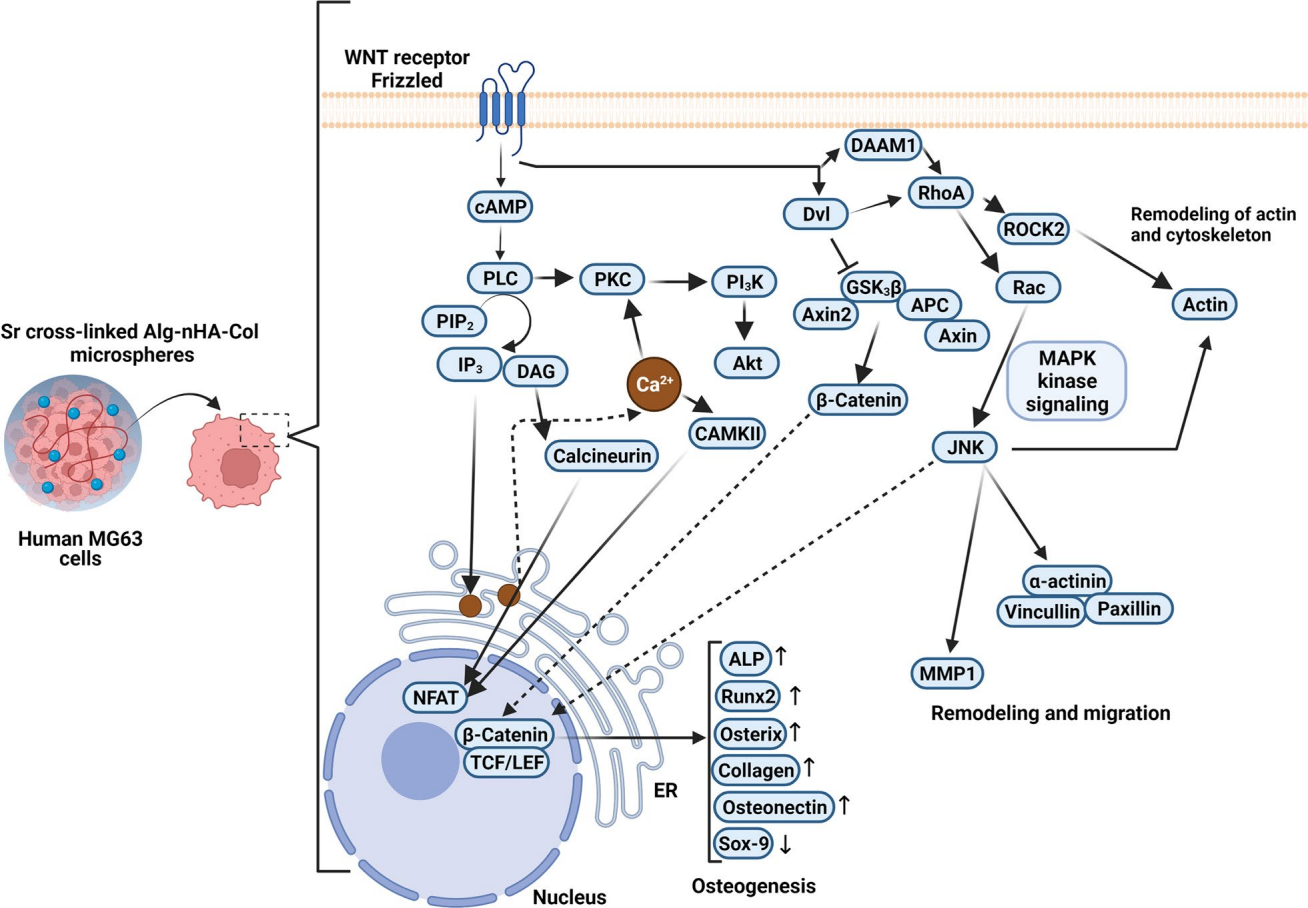

**Supplementary Information:**

The online version contains supplementary material available at 10.1186/s12951-022-01511-9.

## Introduction

Bone tissue engineering aims to fabricate suitable constructs by using both natural and synthetic composites that mimic the physicochemical properties and biological behavior of the osseous system. This strategy is used to circumvent limitations and drawbacks associated with the application of autologous or allogeneic bone grafts such as donor site morbidity, infection, pathogenicity, pain, restricted availability, and immune system reactivity. In this regard, numerous attempts have been directed to accelerate the regeneration of critical-sized bone fractures and restore the structure of non-union fractures [1, 2]. Bone tissue engineering can offer *de novo* therapeutic modalities to support musculoskeletal regeneration using suitable biomaterials, appropriate cell types, and signaling molecules [1–4]. However, the main challenge in front of bone tissue engineering is to provide a 3D niche to dictate suitable functional behavior for each cell type [5–7]. Scaffolds in the form of hydrogels, a cross-linked form of polymer networks with hydrophilic characteristics, have received much attention for tissue engineering applications because of their dynamic functional properties, the best mimicking abilities of the native ECM, encouraging niche for regulating cell attachment, proliferation, cell-matrix interactions, cell to cell communications and corresponding functions [8, 9]. Among the hydrogel-based scaffolding systems, cell-laden hydrogels are the most recent and popular choice as bio-carriers for site-specific cell delivery within the body [10]. These cell-laden hydrogel systems can address several challenges associated with conventional scaffolds, such as the inability to control the complex cellular interactions in the scaffolds, the limited oxygen delivery and mass transfer in large 3D cell and tissue engineering, and heterogeneous cell density [11, 12]. It is suggested that biomaterials without interfering impact on cell homeostasis can be used within hydrogels (microspheres) to promote the viability and functionality of enclosed cells. Furthermore, it seems that the supporting capsules should possess proper mechanical strength and stability to easily permit the reciprocal exchange of nutrients and by-products [13]. To this end, natural anionic polysaccharides, like Alg, are commonly used for encapsulation processes. Regarding suitable biocompatibility and rapid ionic gelation property with divalent cations, alginate-based scaffolds can be appropriately tuned for different 3D-cell culture systems [14–16]. To crosslink an alginate solution, chelating cations are applied to maintain non-covalent bonds within the polysaccharide chains to constitute 3D gel networks [17, 18]. This feature is highly dependent on both the cation and the alginate block structure, and decreases in the following order: Pb [Lead]> Cu [Copper] > Cd [cadmium] > Ba [Barium] > Sr [Strontium ] > Ca [Calcium] > Co [Cobalt]> Ni [Nickel]> Zn [Zinc] > Mn [Manganese] (except Mg^2+^) [19, 20]. Calcium is a common divalent cation, used as a cross linker to fabricate a 3D microenvironment for encapsulation of different cell types. The close ionic interaction of calcium ions carboxyl groups of guluronic acid residues causes the formation of a relatively 3D matrix composed of an Alg backbone [21, 22]. In recent years, barium has frequently been used as cross-linking ion in alginate hydrogels for cell encapsulation purposes, crosslinking the alginate in a similar mechanism in an egg-box model [23–25]. Strontium is one of the essential trace elements in bone structure. Moreover, this element can induce bone matrix ossification via the stimulation of osteoblasts and tethering of osteoclast activity [26–28]. As a correlate, the appropriate selection of certain ions with significant biological effects paves a way for *in vivo* tissue regeneration. Despite several disadvantages like limited bioactivity and relative appropriate physicochemical properties, Alg scaffolds are at the center of attention for the fabrication of engineered osteochondral grafts. In bone engineering, the introduction of inorganic components like HA is touted as an alternate to modulate the mechanical values and bioactivity of Alg [29–31]. Related to the absence of suitable binding sites and excessive anionic charge, Alg alone cannot promote cell adhesion [4, 31, 32]. To overcome these pitfalls, Gel was used in alginate-HA microcapsules to improve osteoblasts' adhesion and growth [29, 31]. However, Gel is continuously released into the medium in the early stages of the cell culture period. Besides, Gel is a denatured form of collagen and possesses minimum attachment sites for cells than that of collagen. Therefore, these values have led to a limited application of Gel in Alg-based hydrogels. Collagen is the main bone ECM protein, facilitates aligned mineralization and simultaneously provides different motifs for cell attachment. Collagen has prominent biodegradability and biocompatibility with negligible immuno-privileged activity after the exposure of telopeptides [33, 34]. However, pure collagen fibers are not eligible enough to accelerate bone formation capacity [35]. In our previous study, Alg-nHA-Col microcapsule cross-linked by Ca^2+^ could provide an appropriate osteogenic building block [36].

Here, we hypothesize that the development of encapsulated osteoblasts inside the Alg-nHA-Col microspheres using three different gelling ions (Ca^2+^, Ba^2+^, and Sr^2+^) can result in varied osteogenic potential by engaging certain effectors related to Wnt signaling pathway. To this end, cell-enclosed microcapsules were developed using various gelling ions (Ca^2+^, Ba^2+^, and Sr^2+^) and their impacts on osteogenic potential were monitored. The influence of crosslinkers was evaluated on the physical-chemical characteristics of the hydrogel as well as microencapsulated cell proliferation, and alkaline phosphatase activity as a well-known indicator of bone formation, and mineralization. Besides, we monitored the expression of different effectors associated with the Wnt signaling pathway along with the protein levels of osteogenesis-related factors. Due to a better osteogenic microenvironment obtained from strontium cross-linked Alg-nHA-Col hydrogels, rat osteoblasts were encapsulated in the strontium cross-linked microspheres and then transplanted into rat cranium with critical size defect to evaluate bone formation by micro-CT analysis and histological examination. To the best of our knowledge, this is the first study to use the strontium cross-linked Alg-nHA-Col hydrogel microcapsule as a high-performance osteogenic microenvironment for modular bone tissue formation.

## Results and discussion

### Influence of crosslinking agent on the microstructure of Alg-nHA-Col composite hydrogel

**Fig. 1:**
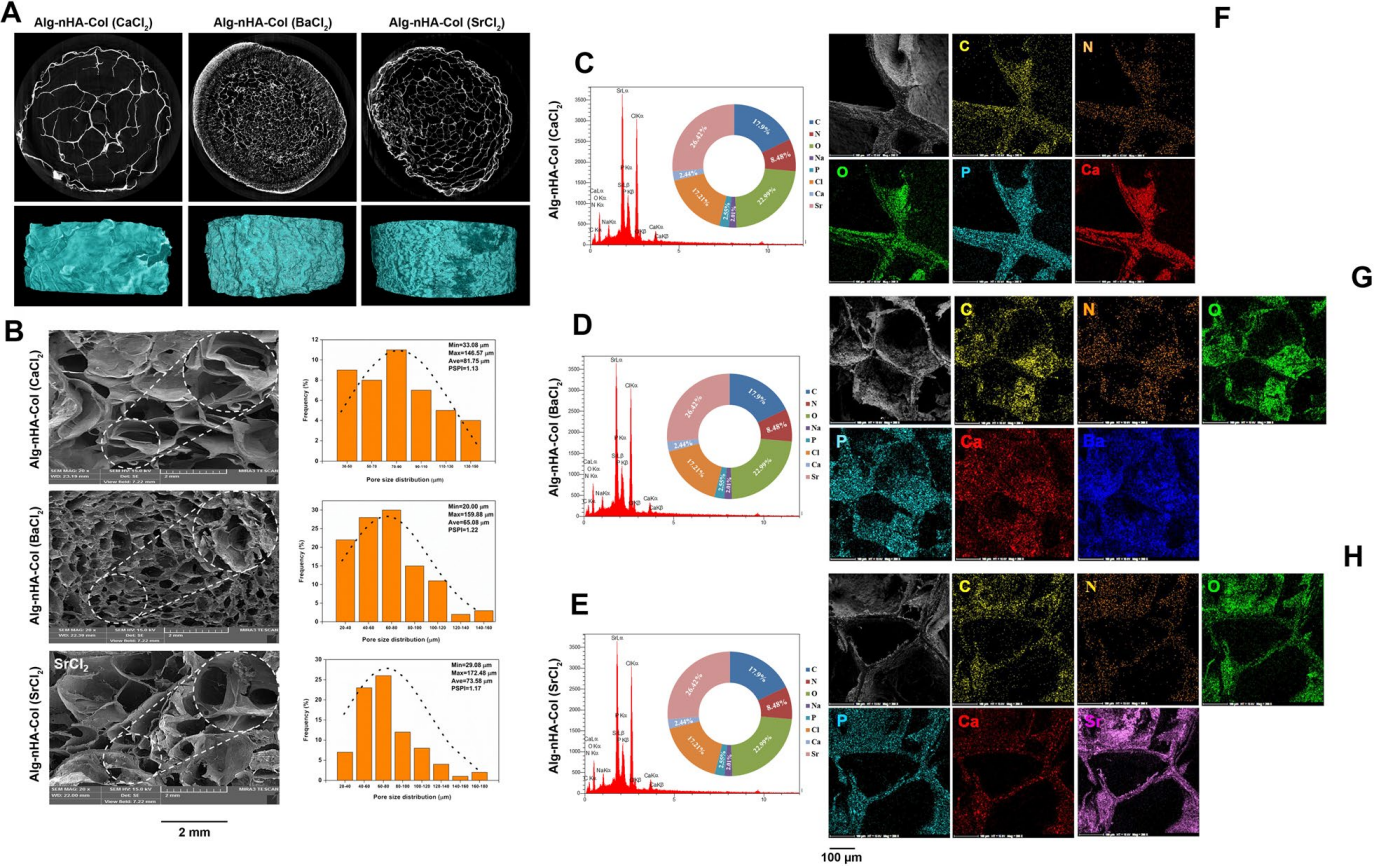
Influence of crosslinking agent on the microstructure of Alg-nHA-Col composite hydrogel. **A** Micro-CT imaging of cross-linked Alg-nHA-Col hydrogels was used for detecting the morphometrical properties. **B** Representative FESEM images of cross-sectional surface morphology of the composite scaffolds reveal pore size distribution. Scale bars are 2 mm. **C**, **D** According to EDX analysis, the type, amount, and how the distribution of the elements on the surface of scaffolds could be perused. **F**–**H** Dot mapping analysis images indicate the distribution of elements within hydrogel lattice

Scaffold morphology is a critical issue with prominent effects on cell migration, attachment, survival, and ECM synthesis in a 3D niche [37]. Sufficient porosity and pore interconnectivity provide a certain microenvironment to promote cell migration, vascularization, proper transport of nutrients and gases, and removal of waste materials. Besides, these structures are resistant to external loading stresses [38, 39]. Micro-CT was used to evaluate scaffold morphometrical properties such as open and closed porosity as well as total porosity of hydrogels (Fig. 1A and Table 1). Regarding Table 1, the total porosity values for Alg-nHA-Col cross-linked by Ca^2+^, Ba^2+^ and Sr^2+^ were 92.1, 75.37, and 82.17% respectively.

**Table 1:** Morphometrical properties of Alg-nHA-Col hydrogels cross-linked by different crosslinkers

Component	Open pore (%)	Closed pore (%)	Porosity (%)	Average pore size (µm)	Gel fraction (%)
Alg-nHA-Col (CaCl_2_)	92.1	0.0018	92.1	81.75	57.98 ± 2.23
Alg-nHA-Col (BaCl_2_)	73.16	2.21	75.37	65.08	33.28 ± 0.76
Alg-nHA-Col (SrCl_2_)	82.16	0.0135	82.17	73.58	37.55 ± 1.08

The cross-sectional surface morphology of the fabricated scaffolds was visualized by SEM (Fig. 1B). Data revealed an average pore size of 81.75 ± 32.83 μm in hydrogels cross-linked with Ca^2+^. In contrast, Sr^2+^ and Ba^2+^ ions diminished the average pore size, to 73.58 ± 30.77 and 65.08 ± 30.93 μm, respectively. As shown in Fig. 1B, Alg-nHA-Col hydrogel cross-linked by Ba^2+^ exhibited the lowest pore size compared to other groups. Smaller pores increase specific surface area and participate in the regulation of cell aggregation and proliferation. Of note, the exogenous hypoxic condition is a challenging issue in such types of hydrogels [40]. Osteoblasts possess a diameter of 10–50 µm [41] and prefer larger pores to accelerate the formation of the mineralized bone matrix after transplantation. Of note, restricted pore size and lack of appropriate space for cell migration can lead to less cellular activity [42]. A relatively similar pore size polydispersity index (PSPI) was obtained for different samples. Cross-linkers had no considerable effects on scaffolds homogeneity. Encapsulation of cells within the hydrogels with smaller pore sizes provides hypoxic conditions, leading to the promotion of chondrogenesis instead of osteogenesis [43]. These features indicate that hydrogels with appropriate pore size and interconnectivity are an essential item in the development of engineered transplants for bone regeneration. EDX and dot mapping analysis of Alg-nHA-Col hydrogel cross-linked with the various metal ions are reported in Fig. 1C-D. According to data, the type, amount, and how the distribution of the elements on the surface of samples can be perused. Alg gelation is initiated following the interaction of divalent cations interact with residues in blocks G. From a molecule's point of view, divalent ions replaced Na^+^ to constitute stable Gel after being exposed to the sodium Alg solution. The affinity of alginate for divalent ions has been shown to decrease in the following order: Ba^2+^  > Sr^2+^  > Ca^2+^ [23]. According to our results, the atomic percentage of Ca^2+^, Sr^2+^, and Ba^2+^ ions in the cross-linked hydrogels were obtained at 9, 26.4, and 55.3% respectively. Furthermore, Ba^2+^ cations have maximum surface charge density with significant interaction with carboxyl and hydroxyl residues in the polysaccharide chain [23]. It can easily be found that the presence of barium ions was the same extent expected on the hydrogel surface, producing a tighter arrangement than others. Notably, the pore size is smaller in Ba-cross linked hydrogel without a prominent interaction with the Alg and gelation capacity [44, 45]. Based on dot mapping analysis (Fig. 1F–H), it is observed the favored blending of Col and nHA with the Alg inside the hydrogel structure.

### Fourier-transform infrared spectroscopy (FT-IR) characterization

**Fig. 2:**
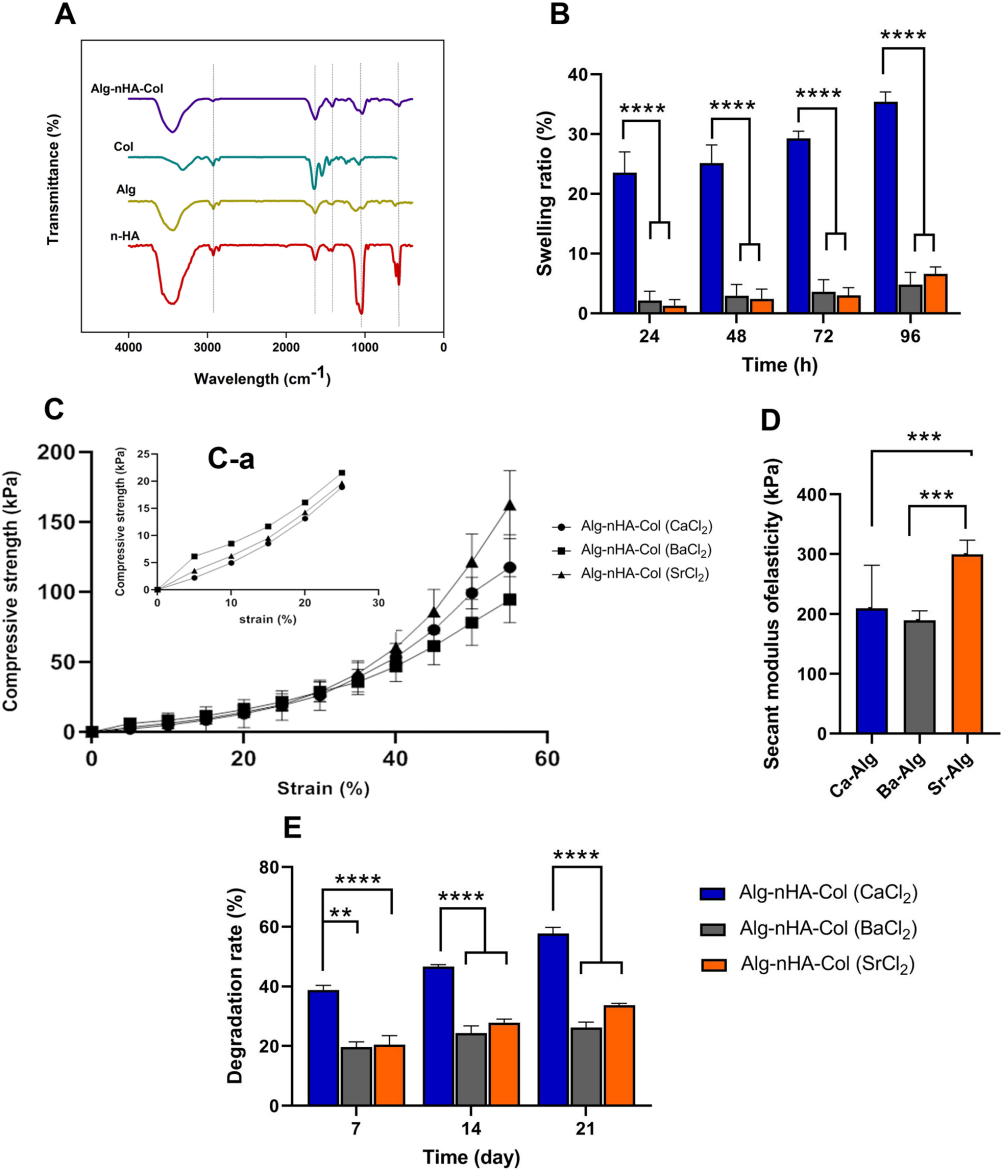
**A** FT-IR spectra of nHA, Alg, and Col components as well as their mixture of Alg-nHA-Col. **B** Influence of crosslinking agent on the swelling ratio of Alg-nHA-Col composite hydrogel during 96 h incubation periods in PBS solution (n = 3). **C** The compressive strength-strain curve for various hydrogel compositions at strain 60%, **C**, **a** compressive strength-strain curve for various hydrogel compositions at strain 30%. The error bars indicate ± standard error, n = 8. **D** Secant modulus of elasticity of the hydrogels calculated from the Compressive strength–strain at strain 60% could be tuned from 200 to 300 kPa. The degradation rate of hydrogels in PBS-lysozyme solution during 28 days (n = 3). (**p < 0.01; ***p < 0.0001; and ****p < 0.00001)

FT-IR was done to monitor functional groups after hydrogel synthesis (Fig. 2A). Intensive absorption bands of n-HA at 560 and 600 cm^−1^ and 1000–1100 cm^−1^ were attributed to phosphate groups [46]. For the OH group, a broad peak was formed at 3200–3500 cm^−1^. Intensive peaks associated with CO_3_^2−^ were detected between 1460 and 1530 cm^−1^ [47]. Absorption bands at 1635 cm^−1^, 1419 cm^−1^, and 1050 cm^−1^ correlate with Alg because of asymmetric stretching vibration of the COO group, and elongation of C–O groups [48, 49]. Of note, in the range of 3400–3600 cm^−1^, stretching vibrations related to O–H bonds appeared. In the context of Col structure, peaks at 1645, 1547, and 1237 cm^−1^ belong to type I Amide [C = O], type II Amide [N–H stretching and C-N deformation], and type III Amide III [C-N deformation and N–H stretching], respectively [50, 51]. In general, the FT-IR spectra of blending of components indicated low intensity and a small shift related to pure Alg, Col, and HA. Taken together, mineral and organic phase interaction can lead to peak shift.

### Swelling ratio of Alg-nHA-Col composite hydrogel

The swelling characteristics of a network are integral for biomolecules releasing, biofluids absorption, and distribution of nutrients within the scaffold structure [52]. The degree of swelling of hydrogels is dependent on the pore size, porosity, gel fraction, and stability of the polymer network [53]. Figure 2B shows the effect of different cross-linkers on the swelling behavior of Alg-nHA-Col hydrogel after 96 h incubating the composite gels in PBS (pH = 7.4) at 37 C. Data showed significant differences in the swelling ratio of hydrogel cross-linked with Ca^2+^ compared to the composite hydrogels cross-linked with Ba^2+^ and Sr^2+^ groups (p < 0.0001). The swelling ratio was observed at 23% for Ca-hydrogel after 24 h (*p*Ca-hydrogel vs. Ba and Sr-hydrogel < 0.0001) reaching 25, 29 35% after 48, 72 and 96 h, respectively (*p*Ca-hydrogel vs. Ba and Sr-hydrogel < 0.0001). It is notified that swelling ratio was highly diminished in the presence of barium and strontium cations in contrast to calcium cations (p < 0.0001). In line with this claim, swelling ratio was reduced about 86% and 81% in Ba- and Sr-hydrogel at the end of 96 h.

Ideally, the swelling of polymeric hydrogels occurs at two mass transport phases. At first, the solvent convection is carried out through the pores of the gel matrix. Afterward, the solvent diffuses into the polymer lattice struts [54]. The diffusion capacity correlates with the ionic size function. When calcium-cross-linked Alg hydrogels are incubated in phosphate buffer saline [PBS; pH = 7.4], Na^+^ ions can bond with –COO^−^ groups in an Alg structure of the medium via an ion-exchange process with Ca^2+^. Therefore, –COO^−^ groups’ electrostatic repulsion is accelerated by time, leading to the relaxation of the Alg chain and swelling. Compared to Ca^2+^ with ionic radii of 0.97 Å, Ba^2+^ and Sr^2+^ possess an ionic radius of 1.35 and 1.26 Å, respectively. Commensurate with these descriptions, the mentioned ions can occupy large-sized spaces between the Alg molecules causing a tightly-regulated arrangement within the intermolecular chain. Consistently, the exchange of Ba^2+^ ions with Na^+^ ions within hydrogel and elimination of insoluble barium phosphate leads to a low swelling ratio (Fig. 2B).

### Mechanical properties of hydrogels

One of the most important properties of scaffolds in hard tissue engineering applications is their mechanical properties. Adequate mechanical values have an indispensable role in injured tissue regeneration and the maintenance of physical form. It is thought that the binding and proliferation capacity of each cell and juxtacrine interaction of cells with ECM can be efficiently modulated when the stiffness of substrate is, but not completely, at the amount of natural tissue [53, 55]. Ideally, hydrogels possess low density with an interconnected lattice, which can be considered cellular solids. The compressive strength curve of cellular solids (Additional file 2: Figure S2) is distinguished by three segregated zones of linear elastic, collapse plateau, and densification [56]. An impressive feature of scaffold that can be affected mechanical properties is porosimetry, including values of pore volume, pore size, and interconnectivity, together with additional information such as bulk density and total porosity [57]. In this study, the influence of different crosslinking agents on the mechanical property of Alg-nHA-Col hydrogels was evaluated by measuring the compressive stress–strain profiles at a constant stretching velocity of 2.0 mm min^−1^. In addition, the secant modulus of elasticity of hydrogels was determined at 60% strain. From the curve change trend in Fig. 2C, it is evident that the deformations of all groups of hydrogels moderately increased as stress increased. An elastic interval with the strain range of 0–30% (Fig. 2C–A) was chosen from the stress–strain curve to assess the effect of microstructure characteristics on mechanical properties. At mentioned strain range, Ba-crosslinked Alg-nHA-Col indicated better compressive strength when compared with other groups. It can be concluded that the pre-strain mechanical properties until 30% rely upon microstructure properties of the hydrogels. According to data, cross-linked hydrogel by Ba^2+^ has the lowest mean pore size and porosity (Fig. 1B and Table 1).

Based on the strain range of 30–60% and the different deformation degrees of cross-linked hydrogels, it can be seen that Sr-crosslinked Alg-nHA-Col possesses the strongest elasticity and compressive strength compared to the other hydrogels. As illustrated in Fig. 2D, the secant modulus of elasticity of cross-linked hydrogels with Ca^2+^, Ba^2+^, and Sr^2+^ ions was found to be 210, 190, and 300 kPa, respectively. As a matter of fact, after the collapse of the porous structure and in the densification zone, the polymeric strength network plays a critical role in the stability of the gel lattice. Alg affinity towards cations is exceedingly dependent on both the cation and the sequence of guluronic (G) and mannuronic (M) residues in the polymeric chain. Binding studies have revealed that Ca^2+^ ions bind to either G or MG blocks. Ba^2+^ ions have an affinity to G and M blocks while Sr^2+^ ions only interact with the G blocks. The affinity of Ba^2+^ toward M-blocks of Alg is greater than that of Ca^2+^ and Sr^2+^ [23]. Furthermore, the interaction of divalent cations with G blocks is highly selective and three blocks' stiffness decreases in the order GG > MM > MG [58]. Minimum G block length can support junction formation and this index reduces by enhancing the affinity of ions toward the Alg chain. Hence, attributable to the fact, replacing Sr^2+^ with Ca^2+^ as a well-known crosslinker resulted in a considerable efficiency of ion entrapment and the presence of shortened but more excessive junctions, which lead to an outstanding gel strength [59]. It has been reported that Matrix mechanical properties influence the focal-adhesion structure, the cytoskeleton, differentiation pathway, osteogenic activity, and mineralization [60, 61]. For stiffer hydrogels with moduli higher than 140 kPa, mineral deposition rate and osteogenesis are induced with increasing modulus to 225 kPa. Cells can respond to the stiffness of their surrounding microenvironment and exhibit appropriate biological activity when the polymeric matrix modulus is relatively similar to the native in vivo tissue values [62].

### Degradation rate of Alg-nHA-Col composite hydrogel

One of the indispensable factors to be considered in hard tissue engineering is the degradation rate of scaffolds as it supplies appropriate space for cell ingrowth followed by neo tissue and matrix deposition, which is crucial for the quality of bone tissue regeneration [63]. Here, in vitro degradation rate was measured using PBS solution containing lysozyme for 28 days (Fig. 2E). Our results revealed that different divalent ions as crosslinking agents significantly influenced the degradation behavior over time. The higher degradation rate was observed at 38.8% for Ca-crosslinked Alg-nHA-Col after 7 days (pCa-Alg vs. Ba-Alg and Sr-Alg < 0.0001) reaching 57.7% after 28 days (pCa-Alg vs. Ba-Alg and Sr-Alg < 0.0001). Interestingly, cross-linked hydrogels by barium and strontium cations could slow down the degradation rate in which Ba-crosslinked Alg-nHA-Col and Sr-crosslinked Alg-nHA-Col decreased degradation rate from 19.7% and 20.5% for 7 days to 26.2% and 33.7% at 28 days, respectively. Of note, there was no statistically significant between Ba-crosslinked Alg-nHA-Col and Sr-crosslinked Alg-nHA-Col hydrogels at all incubation periods. The decreased degradation rate may be due to the value of the gel content in the polymeric matrix decelerating the enzyme functionality. According to Table 1, Alg-nHA-Col hydrogels cross-linked by calcium as well-known cross-linkers agents possess the highest amount of gel content when compared to other agents (pCa-Alg vs. Ba-Alg and Sr-Alg < 0.0001). It can be attributed that the value of the gel fraction is in direct proportion to the hydrogel degradation behavior. The higher the gel contents inside the hydrogel leads to the higher the degradation degree. Besides, another parameter that ought to have crucial effects on the pattern of scaffold swelling and degradation is the microstructure features [64]. Higher porosity and large-sized pores within the constructs cause an extra-permeability and faster degradation. Therefore, the degradation rate of cross-linked hydrogels by calcium is much larger among the other groups.

### Surface and cross-sectional microstructure

**Fig. 3:**
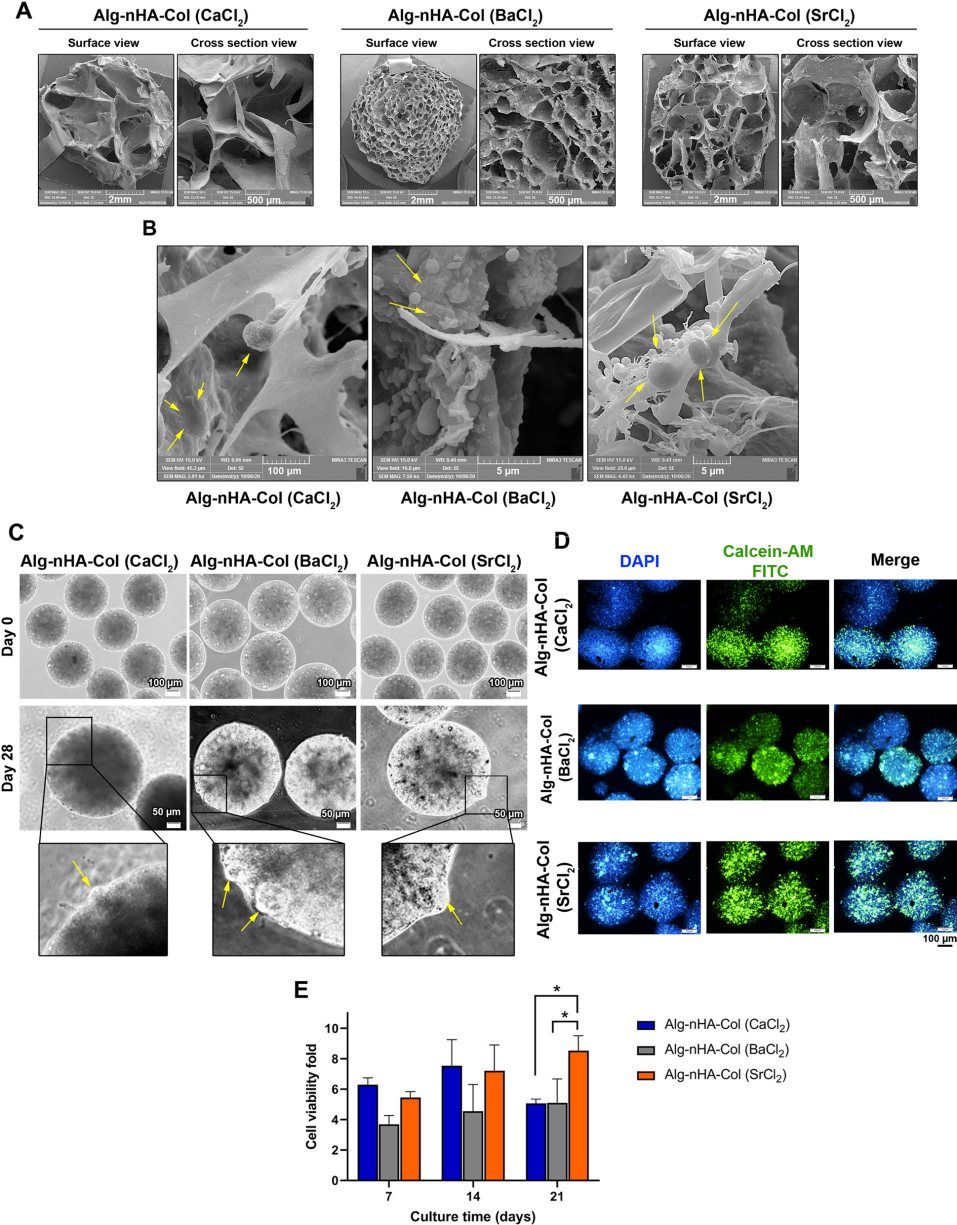
**A** Surface and cross-sectional microstructure of cross-linked hydrogels. Scale bars are 10 µm and 5 µm, respectively. **B** Twenty-one-day incubation of human osteoblasts displayed attachment and morphological adaptation. (Yellow arrows: cells). **C** Bright-field imaging revealed an almost uniform size of microspheres in three groups cross-linked using Ca, Ba and Sr. Scale bars for days 0 and 21 are 100 µm and 200 µm, respectively. Tumefy areas on the periphery of all microsphere types are associated with the cell motility and growth of encapsulated cells. **D**, **E** Cell viability and proliferation of human osteoblast-like MG-63 cells were monitored using Calcein- AM staining and MTT assay after 21 days of microencapsulation. (Live cells: green and nuclei: blue). Significance is indicated (* p < 0.05)

Both surface and cross-sectional morphology of prepared hydrogels were monitored (Fig. 3A). Data showed an irregular pore pattern in the surface and cross-section view of Alg-nHA-Col cross-linked using calcium. Compared to the microspheres fabricated using Ca^2+^, the application of Ba^2+^ yielded irregular pores with a smaller size which can be in surface and cross-section imaging. Similar to Ca-cross-linked hydrogel, a relatively regular pore pattern with a smaller size can be detected in the surface and cross-section imaging of hydrogel prepared using Sr (Fig. 3A). These data showed the porous nature of hydrogel fabricated using different ionic cross-linkers. All hydrogels exhibited lamellae with interconnected egg-box morphology which can provide appropriate sites for cell homing and localization. Regarding the type and nature of the cross-linker, the pattern and size of pores can be different in the final structure. Twenty-one-day incubation of human osteoblasts displayed attachment and morphological adaptation (Fig. 3B). The cells were suitably fattened and acquired new morphology inside the microspheres. Bright-field imaging revealed an almost uniform size of microspheres in three groups cross-linked using Ca^2+^, Ba^2+^, and Sr^2+^ (Fig. 3C). The existence of bulged areas on the periphery of all microsphere types is associated with the cell motility and growth of encapsulated cells.

### Cell viability and proliferation

The viability and proliferation of human osteoblast-like MG-63 cells were monitored using Calcein- AM staining and MTT assay after 21 days (Fig. 3D, E). IF imaging revealed the existence of green-colored Calcein AM cells indicating the viability of encapsulated cells in all three groups after 21 days (Fig. 3D). Based on our data, the intensity and number of Calcein AM/DAPI positive cells were increased prominently in microspheres cross-linked by Sr^2+^ compared to other groups. MTT analysis revealed an enhanced cell survival rate in human osteoblasts encapsulated inside Alg-nHA-Col hydrogel cross-linked by Ca^2+^, Sr^2+^, and Ba^2+^ (Fig. 3E). To be specific, hydrogel microencapsulated by Ca^2+^ as crosslinking agent exhibited a maximum growth rate after the 14 days of cultivation, whereas cell proliferation was achieved to 7.5 fold. However, after that, the cell expansion rate decreased. As seen, microencapsulation by Sr^2+^ divalent ion exhibited an increased cell proliferation fold up to 21 days that the value reached 8.5 fold. Barium cross-linked cell-laden microcapsules showed also a similar proliferation profile with a maximum fold of 5.1. Consistent with the current data, it was suggested that the fabrication of combined titanium-based Sr^2+^ HA led to osteoblast proliferation [65]. Based on our data, the encapsulation of human MG-63 inside Alg-nHA-Col hydrogels cross-linked with Ba^2+^ and Sr^2+^ activated antioxidant enzymes such as GPx compared to the Alg-nHA-Col (CaCl_2_) group (*p* < 0.05). Despite SOD and TAC activity in Alg-nHA-Col (BaCl_2_) and Alg-nHA-Col (SrCl_2_) groups, non-significant differences were obtained as compared to the Alg-nHA-Col (CaCl_2_). Some studies demonstrated that scaffold biophysical properties have a significant impact on cell bioactivities in a 3D environment. Besides, cell morphology and functions have a close relationship with substrate stiffness under circumstances when the levels of chemical signals are not changed. On the other hand, it has been reported that cells can sense microenvironment stiffness and rigidity [66–69]. Hydrogel stiffness can be regulated by changing the crosslinking density, cross-linker type, and molecular weight of the precursors [70–73]. The osteogenic capacity of strontium is associated with elevated bone mineralization and reduced bone resorption. It was suggested that the calcium-sensing receptor (CaR) is an important factor involved in the dynamic growth and functional activity of osteoblasts in the presence of strontium [74]. Based on our findings, the osteoblast-like cells inside Sr cross-linked hydrogel showed higher proliferation and mineralization rates with higher rigidity and stiffness compared to other groups. It was observed that all cell-laden microcapsules demonstrated a reduction trend of cell proliferation after reaching a maximum during the culture period especially in Alg-nHA-Col (CaCl_2_). One reason would be that Ca-Alginate microcapsules had a relatively maximum pore size which can increase cellular activity in the early stages of in vitro conditions. However, the crosslinking ions can be gradually replaced by other cations such as Na during the wash-culture process. Based on swelling and biodegradation rates, it seems that these effects were more prominent in hydrogels cross-linked by Ca^2+^ compared to the other groups. In addition, partial hydrolysis of hydrogel causes an acidic microenvironment due to the production of alginic acid, leading to a reduced survival rate [75]. The fast geometrical changes due to prompt swelling rate and degradation can lead to structural disintegration and loss of cell-to-scaffold interaction and attachment rate [76]. The existence of maximum survival rate in the Sr-crosslinked group is due to an appropriate swelling rate and biodegradability that promote the osteogenic behavior of human osteoblasts.

### Alkaline phosphatase (ALP) activity

**Fig. 4:**
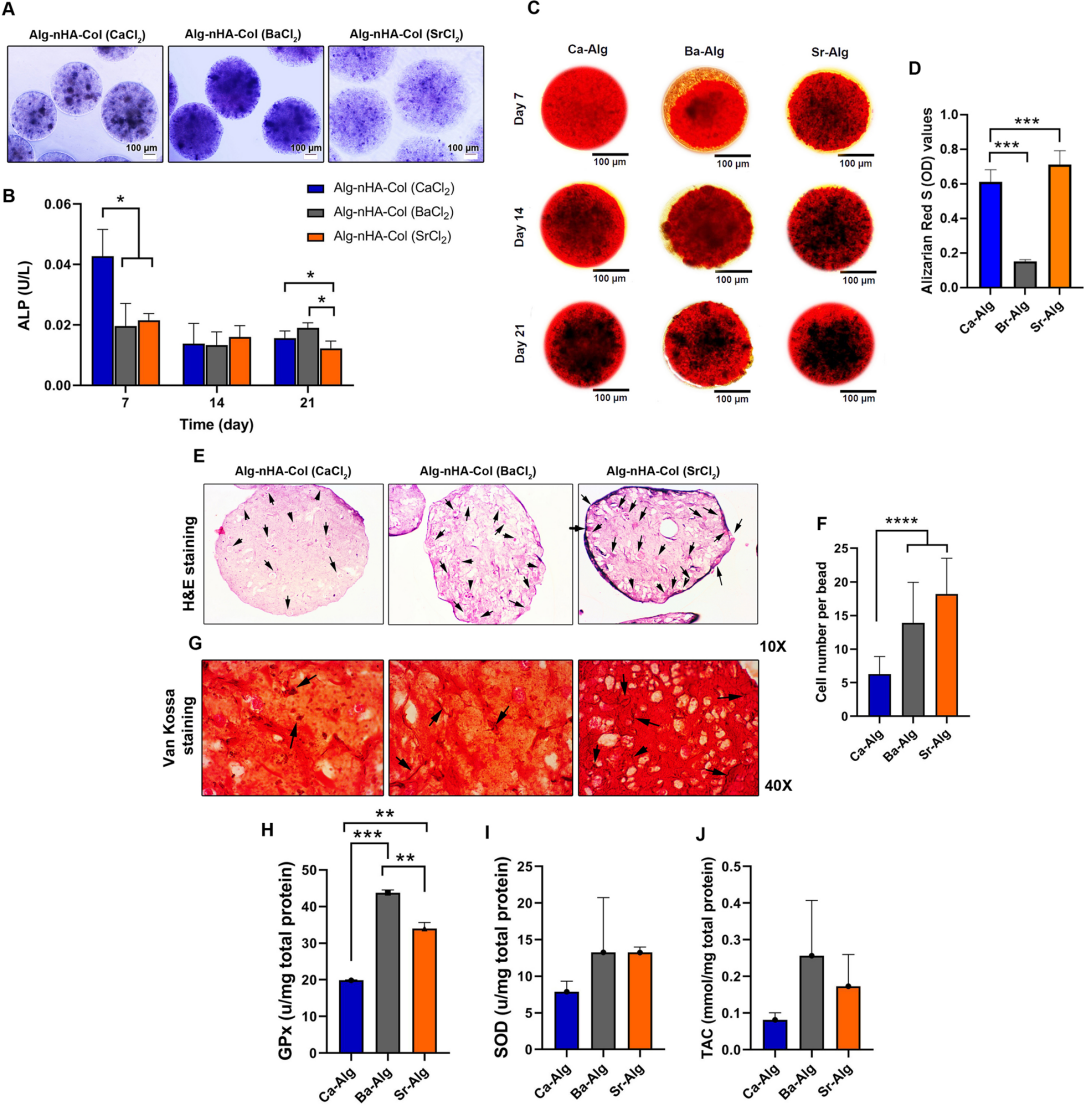
**A** Assessing mineralization and calcified extracellular matrix using Alizarin Red staining revealed that the mineralization rate was more evident in Sr-crosslinked Alg-nHA-Col microspheres related to other groups. **B** ALP staining indicated typical purple spots in Sr^2+^-contained microspheres compared to the other groups (scale bars = 100 µm) and **C** quantification of the ALP activity on days 7, 14, and 21. **D** H&E staining and E) the mean number of MG-63 cells per section of Alg-nHA-Col (SrCl_2_) microspheres was more compared to the Alg-nHA-Col (CaCl_2_) and Alg-nHA-Col (BaCl_2_) groups (p < 0.00001). F) Van Kossa staining revealed the osteogenic capacity of Alg-nHA-Col (SrCl_2_) microspheres to induce calcium deposition. G-I) Monitoring antioxidant capacity of encapsulated MG-63 cells Glutathione peroxidase (GPx; G; n = 3), Superoxide dismutase (SOD; H; n = 3), and Total antioxidant capacity (TAC; I; n = 3) 21 days after cell encapsulation process. * p < .05; ** p<0.01; *** p < 0.0001; and **** p < 0.00001

ALP activity is important for the mineralization of bone and has been considered a marker of osteoblastic activity. The ALP activity levels were observed for cell-laden Alg-nHA-Col microencapsulated by ionic crosslinking with Ca^2+^, Ba^2+^, and Sr^2+^ after 7, 14, and 21 days (Fig. 4A, B). Based on the results demonstrated, ALP activity showed no significant change for encapsulated cells within Ba-Alg hydrogel. By contrast, ALP was reduced in cells exposed to Ca^2+^ on day 7 compared to day 14 and without changes until day 21. For cells grown within the Sr-cross-linked Alg-nHA-Col microcapsules, reduced ALP activity was achieved after day 7 and continued until the endpoint of the experimental period (day 21). The notable point is that ALP can hydrolyze organic phosphate compounds at basic pH, participating in ossification and neo-tissue mineralization. The observations in some studies have suggested that higher ALP activity is integral to ECM remodeling in the presence of osteoblasts before mineralization [77–79]. A substantial role of ALP in mineralization was previously determined with a time-dependent expression of this enzyme along with osteogenesis and ossification of cartilage tissue. It is believed that ALP can be produced in bone and calcifying cartilage and attached to the cell surface or released via secretory vesicles. With the expression of other genes such as Osteonectin in the latter phases, transcription of ALP diminished along with the initiation of mineralization [80]. In other words, ALP increases at the early stages of osteogenesis and this trend is reversed when mineralization is well progressed. Indeed, it is of interest to consider that cell-laden hydrogel by ionic crosslinking with Sr^2+^ possibly improved osteogenic properties over both Ca and Ba-cross-linked Alg-nHA-Col. In the support of current data, Geng and collaborators indicated that Sr^2+^ possesses the superiority to simultaneously induce osteogenesis via HA mineralization and inhibit osteoclastogenesis [81]. Even a combination of Sr with other elements can improve osteogenesis outcomes [65].

### Mineralization and calcium deposition analyses

Key evidence for bone formation is the production of mineralized calcium components by osteoblasts. The mineralization rate was evaluated in vitro by staining the calcium deposits with Alizarin red. It was notified that mineralized Ca^2+^ contents increased in all three groups by time in which Ca^2+^ content reached maximum levels on day 21 compared to 7 and 14-time points (Fig. 4C). Based on our data, the increase of mineralized calcium in microspheres cross-linked with Ba^2+^ showed a minimum Ca^2+^ increase compared to the other groups. By contrast, the mineralization rate was more evident in Sr^2+^-contained microspheres compared with other groups. Of note, we found moderate Ca^2+^ mineralization in microspheres fabricated using Ca^2+^ as a crosslinker. H& E staining showed the distribution of MG-63 cells inside Alg-nHA-Col microsphere parenchyma. According to our data, the mean number of MG-63 cells per section of Alg-nHA-Col (SrCl_2_) microspheres was more compared to the Alg-nHA-Col (CaCl_2_) and Alg-nHA-Col (BaCl_2_) groups (*p* < 0.0001; Fig. 4D, E). These data were in line with the results of MTT and survival assays. Von Kossa staining revealed that the deposition of Ca^2+^ mineralization caused a string-like appearance in Alg-nHA-Col (SrCl_2_) microspheres compared to the other groups (Figure F). Compared to the Alg-nHA-Col (SrCl_2_) group, this pattern was less in Alg-nHA-Col (CaCl_2_) and Alg-nHA-Col (BaCl_2_) groups. According to our findings, Sr^2+^ as a crosslinking agent had a promising ability to encourage bone formation. For this reason, although strontium is one of the essential trace elements in bone structure, the study demonstrated that the activation of osteoblast activity and suppression of osteoclast function can increase bone mass [26]. Both ALP and mineralization results showed the ability of strontium to promote the osteogenic activity of microencapsulated cells within Alg-nHA-col hydrogel *i.e.* their proliferation, mineralization, and, in consequence, bone regeneration.

### Alg-nHA-Col (SrCl_2_) microspheres activated the Wnt signaling pathway of MG-63 cells

We performed PCR array analysis to monitor the expression of different genes related to the Wnt signaling pathway (Additional file 3: Table S1). For this end, MG-63 cells were encapsulated inside Alg-nHA-Col using crosslinkers such as Ca^2+^, Ba^2+^, and Sr^2+^. In this assay, Alg-nHA-Col (CaCl_2_) group was considered the control group, and the expression of genes in other groups was compared to this group. In general, data displayed the majority of genes from the Wnt pathway were up-regulated in the Alg-nHA-Col (SrCl_2_) group compared to the microspheres containing Ba^2+^ and Ca^2+^ (Table 2). According to our data, the incubation of MG-63 cells inside the Alg-nHA-Col (BaCl_2_) and Alg-nHA-Col (SrCl_2_) microspheres reduced the expression of AES compared to the Alg-nHA-Col (CaCl_2_). It has been shown that this gene acts as a repressor to NF-ƙB, indicating the reduction of pro-inflammatory conditions in cells encapsulated with a hydrogel containing Sr^2+^ and Ba^2+^. It could be noted that the expression of AXIN1, APC belonging to Canonical Wnt signaling was significantly down-regulated compared to the Alg-nHA-Col (CaCl_2_) group (*p* < 0.05). Moreover, the expression of AXIN2 (-5.58-fold) decreased in the Alg-nHA-Col (BaCl_2_) group while the incubation of MG-63 cells inside Alg-nHA-Col (SrCl_2_) microspheres induced the transcription of AXIN2 (30.59-fold) compared to the microspheres composed of Ca^2+^ and Ba^2+^ elements (*p* < 0.05). The expression of other genes related to Canonical Wnt signaling pathways such as CSNK2A1, DKK1, CTBP1, CTNNB1, CTNNBIP1, DKK3, DVL1, and 2, FRAT1, FZD1, 2, 3, 4, 5, 6, 7, 8, LRP5, and 6, LEF1, NKD1, PORCN, WIF1, WNT1, 10A, 2, 2B, 3, 4, 6, 7A, 7B, TCF7, SFRP1, SFRP4TCF7L1, and WNT8A was also stimulated in cells encapsulated with Alg-nHA-Col in the presence of SrCl_2_ compared to Alg-nHA-Col (CaCl_2_) group (*P* < 0.05; Table 2 and Additional file 2: Figure S2). Compared to the Alg-nHA-Col (SrCl_2_) group, these genes had fewer expression rates or were down-regulated when MG-63 cells were maintained inside the Alg-nHA-Col (BaCl_2_) microspheres. These features show that Alg-nHA-Col cross-linked with SrCl_2_ can efficiently up-regulate the expression of several genes related to the Canonical Wnt signaling pathway. We also monitored the expression of different genes associated with planar and tissue polarity. These phenomena refer to morphological adaptation of cells and subcellular localization of organelles in response to culture conditions. In this line, data demonstrated that the expression of DAAM1, DVL1, DVL2, MAPK8 (JNK1), NKD1, VANGL2, WNT9A, AXIN2, FZD2, 3, 5, and 6 were significantly up-regulated in the Alg-nHA-Col (SrCl_2_) group. By contrast, both genes such as RHO, and PRICKLE1 were down-regulated from the same signaling pathway. Again, most of the genes related to planar gen and tissue polarity were down-regulated in the Alg-nHA-Col (BaCl_2_) group compared to the Alg-nHA-Col (SrCl_2_) group. In genes with an up-regulated pattern, the changes were not as similar to the Alg-nHA-Col (SrCl_2_) group. These data confirmed that cytoskeletal remodeling and adaptation of human MG-63 cells were more prominent in Alg-nHA-Col hydrogel cross-linked with Sr. The expression of genes such as CTNNB1, DKK1, WNT1, and WNT3A were significantly induced in the Alg-nHA-Col (SrCl_2_) group compared to other groups, showing enhanced differentiation rate. It is believed that the activation of these genes can help the encapsulated cells acquire functional maturation. The modulation of distinct genes in the Wnt signaling pathway can alter cell growth and proliferation rate. For example, we showed that the transcription of CCND1, CCND2, CTBP1, CTNNB1, CTNNBIP1, FZD3, JUN, LRP5, MMP7, MYC, PPARD, and WNT3a has been significantly increased in cells cultured inside Alg-nHA-Col (SrCl_2_) hydrogel compared to the Alg-nHA-Col (CaCl_2_) group, leading to the stimulation of cell proliferation (*P* < 0.05). As such, Alg-nHA-Col (SrCl_2_) hydrogel increased the expression of genes (DKK1, LRP5, LRP6, WNT1) participating in cell migration (*p* < 0.05). The intensity of these changes was less in Alg-nHA-Col (CaCl_2_) and Alg-nHA-Col (BaCl_2_) groups. According to PCR array analysis of the Wnt signaling pathway, it is suggested that 21-day culture of human MG-63 cells inside the Alg-nHA-Col microsphere cross-linked with Sr^2+^ induced the expression of different genes higher than that of other groups. It seems that cells in the Alg-nHA-Col (SrCl_2_) group can enter suitably into the developmental processes such as migration, proliferation, and hemostasis, showing the superiority of Sr cross-linked Alg-nHA-Col in the osteogenic capacity of human MG-63 cells.

**Table 2:**
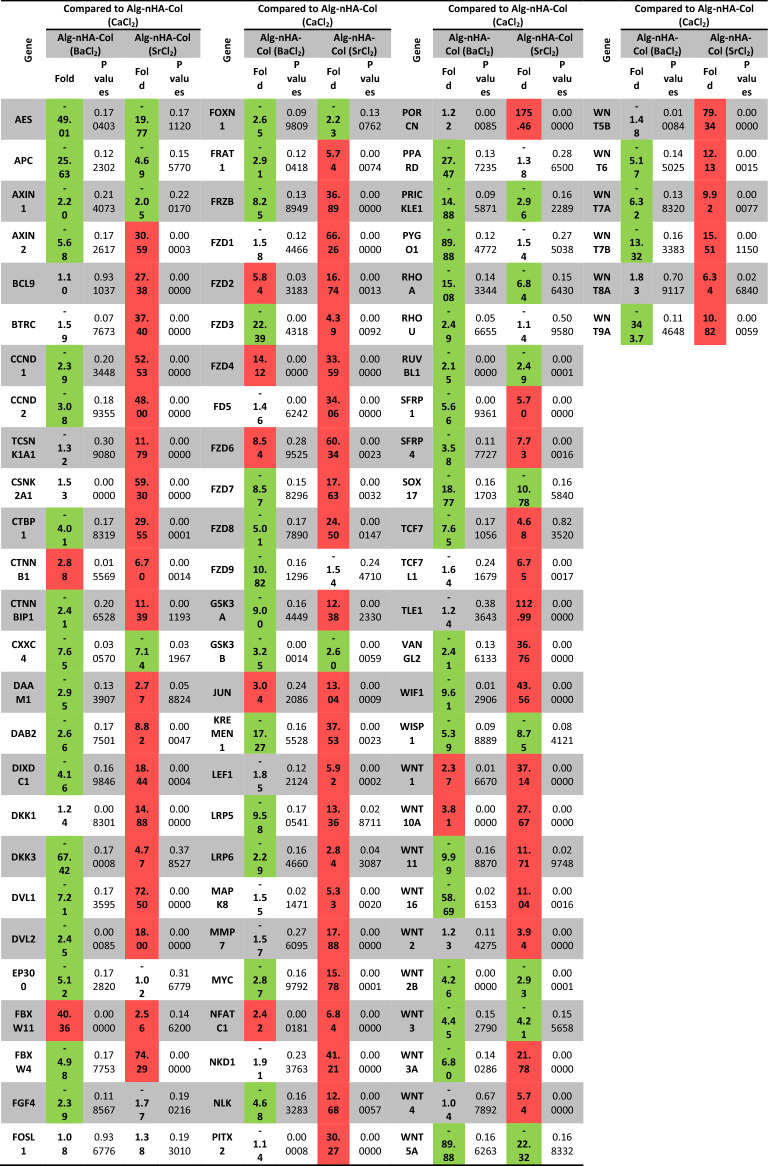
Monitoring the expression of genes related to Wnt signaling pathway in MG-63 cells cultured for 21 days inside the Alg-nHA-Col hydrogels cross-linked using Ca, Br, and Sr

Fold-change and fold-regulation values greater than 2 are indicated in red; fold-change values less than 0.5 and fold-regulation values less than -2 are indicated in green. The p values are calculated based on a Student’s t-test of the replicate 2^(− Delta CT)^ values for each gene in the control group and treatment groups, and p values less than 0.05 are indicated in red

### Western blotting

**Fig. 5:**
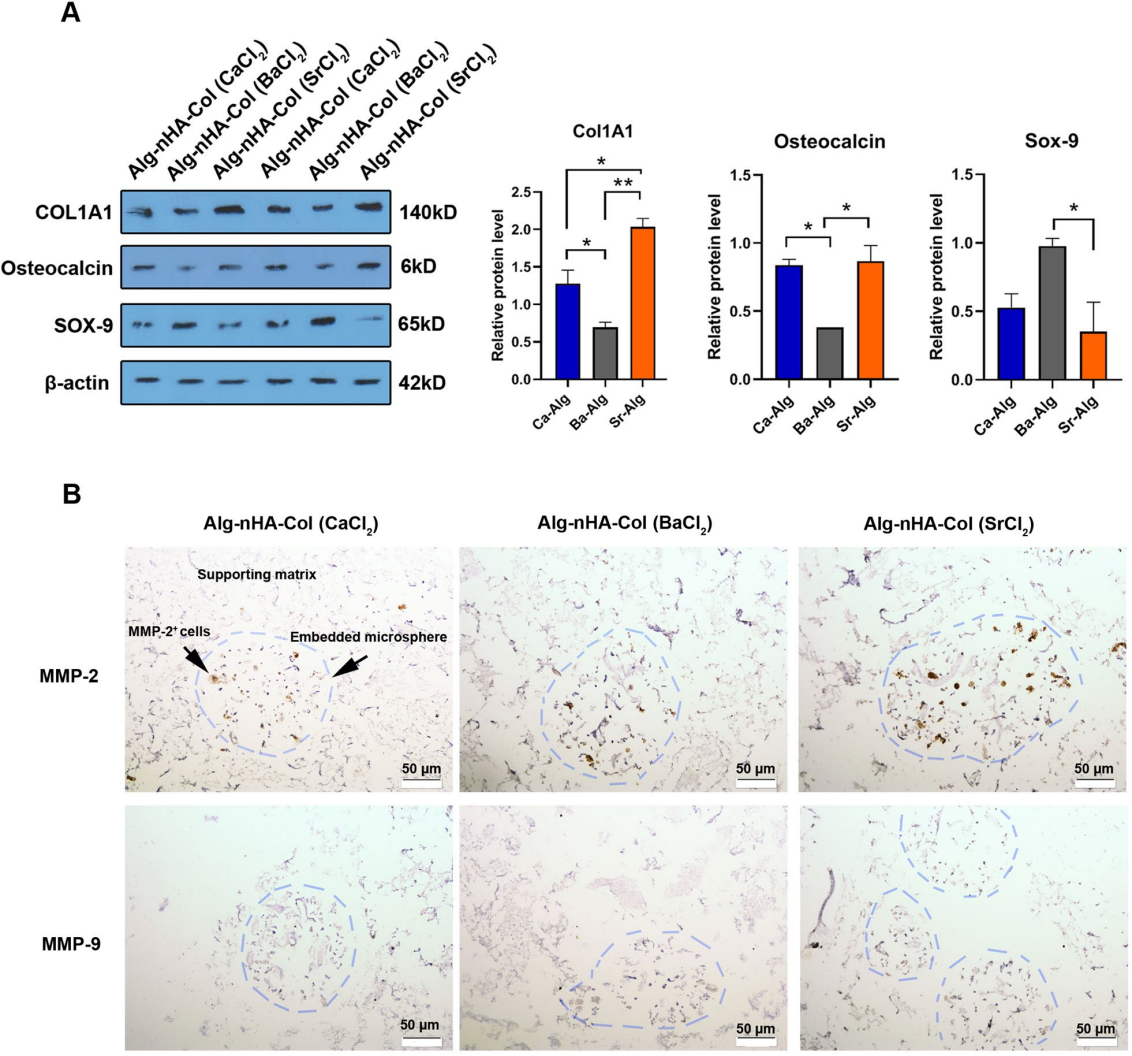
Western blot analysis for the detection of protein levels of ColA1, osteocalcin (OCN), and Sox-9 on 21 post-encapsulation days. The results revealed that the encapsulation of human MG-63 cells inside Alg-nHA-Col microspheres could alter osteogenesis-related factors such as ColA1 and osteocalcin. The β-actin-specific band is used as a standard. One-way ANOVA analysis and Turkey post hoc test (*p < 0.05; ** p < 0.01)

Proteomic analysis revealed that the encapsulation of human MG-63 cells inside Alg-nHA-Col microspheres can alter osteogenesis-related factors such as ColA1 and OCN (Fig. 5A). Data exhibited that 21-day incubation of human MG-63 cells inside Ca and Sr cross-linked Alg-nHA-Col microspheres significantly increased protein levels of ColA1 and osteocalcin compared to the microspheres cross-linked with Ba^2+^ (p < 0.05). Based on our data, the application of Sr^2+^ had superior effects to promote collagen synthesis in comparison with Ca^2+^ (p < 0.05). By contrast, we found a non-significant difference in protein levels of Osteonectin in groups containing Sr^2+^ and Ca^2+^. Among different ionic cross-linkers used, it was suggested that the application of Ba^2+^ increased Sox-9, a chondrogenic factor, and may lead to delayed osteogenesis in encapsulated cells. Taken together, the fabrication of Alg-nHA-Col microspheres using cross-linker Sr^2+^ induces osteogenic properties of human osteoblasts. In a study conducted by Geng and co-workers, they found that titanium coated with Sr-HA can stimulate the synthesis of osteoblast-related factors such as OCN and ColA1 and increase the attachment of cells via the promotion of surface integrin receptors, leading to osteogenesis [82].

### Sr-based Alg-nHA-Col microspheres induced migration of encapsulated MG-63 cells

IHC staining revealed an increased MMP activity in cells encapsulated inside Alg-nHA-Col microspheres using Sr^2+^ compared to other groups (Fig. 5B). Data showed an increase of MMP-2 in Sr-based microspheres when compared to Ca^2+^ and Ba^2+^-based microspheres. Unlike MMP-2 content, the incubation of MG-63 cells inside microspheres cross-linked using Ca^2+^, Ba^2+^ and Sr^2+^ did not yield prominent differences in the levels of MMP-9. These data indicated that the type of cross-linker can affect cell migration via the production of certain types of MMPs in MG-63 cells encapsulated within the Alg-nHA-Col matrix.

### Micro-CT measurements and histological examination of calvarial defects

**Fig. 6:**
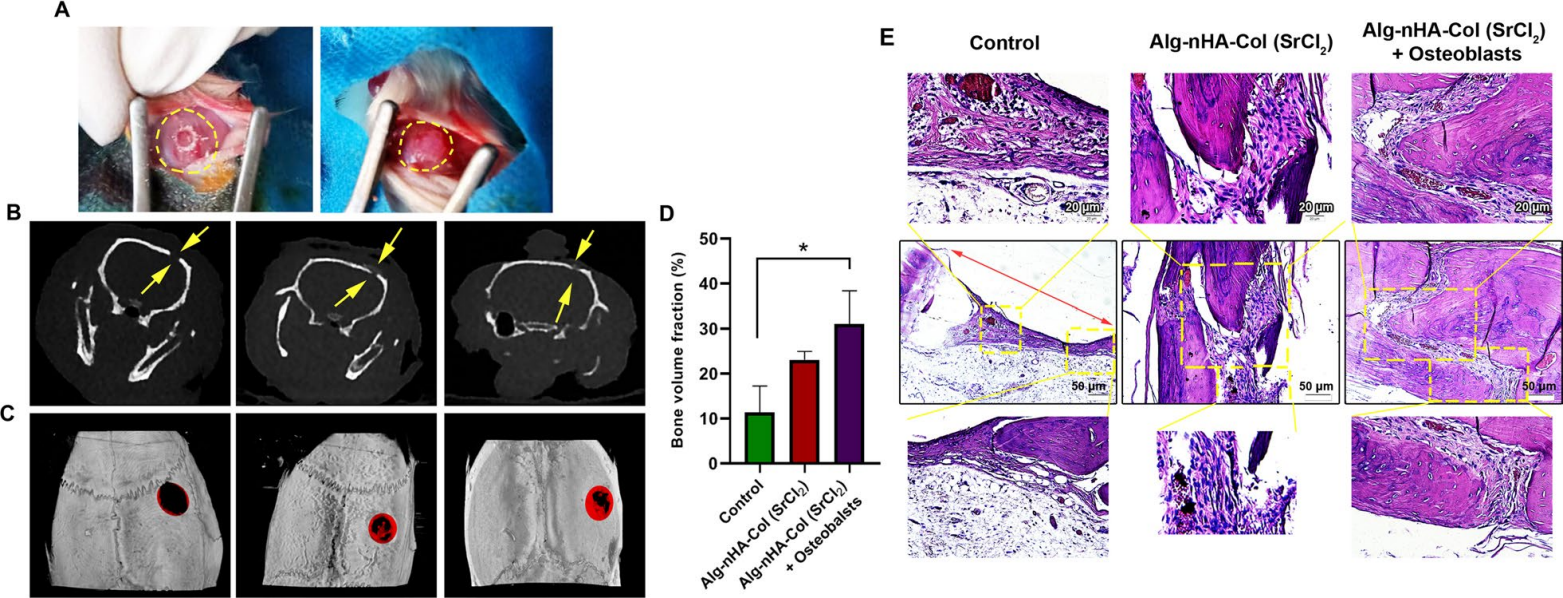
Micro-CT measurements and histological examination of calvarial defects. **A** Implantation of osteoblasts encapsulated inside Alg-nHA-Col microspheres cross-linked with Sr^2+^. **B**, **C** CBCT and micro-CT scanning reveal de novo bone formation in the calvarial defect in the rat model to assess the osteogenic capacity of osteoblasts encapsulated inside Alg-nHA-Col microspheres cross-linked with Sr^2+^ after 8 weeks. Regenerated bone is pseudo-colored red. **D** Quantitative analysis of bone formation based on micro-CT images. Significance is indicated (p < 0.05). n = 4 for each group. **E** Microanatomical demonstration of repair of critical-size defects in the rat calvaria after 8 weeks of transplantation stained with **H **& **E**

Based on the various in vitro assays, we selected Alg-nHA-Col hydrogel with Sr^2+^ cross-linker for in vivo assay. To this end, a 5 mm critical-sized calvarial defect was used in the rat model to assess the osteogenic capacity of rat osteoblasts encapsulated inside Alg-nHA-Col microspheres cross-linked with Sr after 8 weeks (Fig. 6A). Cone-beam computed tomography systems (CBCT) revealed the lack of notable de novo bone formation along the margins of defect areas in the control rats (Coronal slices; Fig. 6B). Data showed a thin radiopaque line (osseous flap) extending from margins toward the center of defects in rats that received Alg-nHA-Col and/or Alg-nHA-Col plus rat osteoblasts. In contrast to the Alg-nHA-Col group, the defects were near to completely closed after implantation of Alg-nHA-Col containing rat osteoblasts. The comparative quantification of bone formation in three groups using micro-CT indicated that the mean newly formed bone volume reached maximum levels in defects filled with Alg-nHA-Col plus rat osteoblasts compared to the control defects without hydrogel (*p* < 0.05; Fig. 6C, D). Despite the increase of bone formation volume in defects filled with the combination of hydrogel and osteoblasts, no statistically significant differences were achieved related to the Alg-nHA-Col group (*p* > 0.05). Taken together, the density of de novo formed bone was accelerated in a critical-sized calvarial defect when the combination of rat osteoblast and Alg-nHA-Col was used.

H & E staining confirmed the progression of defect margins toward the center zone in groups filled with hydrogel alone or in combination with osteoblasts (Fig. 6E). The transplantation of hydrogel plus osteoblasts enhanced osteogenesis of defect margins in which new bone formation areas can be detected. Between the edges of extending margins, a small content of compressed fibrotic tissue can be detected (Fig. 6E). By contrast, the content and volume of connective tissue were larger in rats that received hydrogel only. In the control rats, the margins failed to progress, and only loosely tissue filled the space.

## Conclusions

To sum up, 3D scaffolds are still the most suitable substrates for mimicking the in vivo condition for expanded cells. We proposed that the type of cross-linkers can change the osteogenic capacity of osteoblast within Alg-nHA-Col microspheres. Based on our data, the fabrication of cross-linked microspheres using Sr^2+^ is supposed to appropriately induce the osteogenic activity of human and rat osteoblasts in in vitro and in vivo conditions compared to the other two ions, Ca^2+^ and Ba^2+^. It seems that Sr^2+^cross-linked Alg-nHA-Col microspheres are functionalized matrices to alter the expression of effectors associated with osteogenic capacity. Osteoblasts encapsulated inside the Sr^2+^ cross-linked Alg-nHA-Col microspheres preferably exhibit osteogenic potential after the modulation of the Wnt signaling pathway. Along with in vitro data, we showed the eligibility of Sr-cross-linked microspheres to restore the function of injured bone areas in critical-sized calvarial defects. These findings introduce Sr-cross-linked Alg-nHA-Col composite hydrogel as an advisable high-performance 3D platform for bone tissue engineering applications. The present study faces some limitations and needs further attention. For example, we did not measure ion release performances of Sr^2+/^Ba^2+^/Ca^2+^ cross-linked Alg-nHA-Col hydrogels over time. Evaluation of ion release can give us valuable data about hydrogel behavior and other values which are important in an engineered scaffold.

## Materials and methods

### Materials

Human MG-63 cell line was provided by the Iranian National Cell Bank (Pasture Institute; Iran). Pen/Strep, FBS, PBS, DMEM/LG, and 0.25% Trypsin–EDTA solution were obtained from Gibco (UK). Sodium alginate (I-1G, high content of guluronic acid, and MW: 70 kDa) was purchased from Kimica (Tokyo, Japan). BCIP/NBT alkaline phosphates color development kit, lysozyme, nano-hydroxyapatite, *p*-NPP, barium chloride, strontium chloride, DMSO, and MTT powder were purchased from Sigma-Aldrich. Alizarin Red S, calcium chloride, magnesium chloride, Tris-Based, sodium chloride, paraformaldehyde, and trisodium citrate dehydrate were obtained from Wako Pure Chemical Corporation (Osaka, Japan). Collagen type I was obtained from SBPE Company (Tabriz, Iran). SOD, TAC, and Superoxide dismutase assay kits were purchased from Randox (Crumlin, United Kingdom). Sodium sulfate was obtained from Merck (Germany). RIPA lysis buffer kit, HRP-conjugated anti-IgG, B-actin, COL1A1, COL2A1, OCN, and SOX-9 antibodies were purchased from Santa Cruz Biotechnology (Dallas, TX, USA).

### Hydrogel preparation

The hydrogel scaffold containing sodium Alg, nHA, and Col were prepared by ionic crosslinking of the polymer solution as described in our previous research [36]. Briefly, Alg (1%, w/v) and nHA powder (0.5%, w/v) sterilized by 70% ethanol were dissolved in calcium-free Krebs Ringer HEPES-buffered saline (CF-KRH, pH = 7.2–7.4) for 24 h under a laminar hood. Type I Col solution (pH = 6.5) was sterilized by chloroform at 4 ^◦^C overnight. The homogenous solution of Alg-nHA-Col was prepared by mixing the pre-cooled Col solution with the Alg-nHA solution having a final solution with a concentration of 0.5% (v/v) collagen. Solutions containing divalent cations (CaCl_2_, BaCl_2,_ and SrCl_2_) with a concentration of 0.2 M were used for composite gelation.

### Scanning electron microscope (SEM)

Hydrogel samples were frozen and lyophilized for 48 h. The cross and surface sections of freeze-dried samples were cut by a surgical blade, sputtered with gold, and SEM images were acquired on a Field Emission Scanning Electron Microscopy (FESEM; MIRA3 TESCAN) in conjunction with an energy-dispersive X-ray spectrometer (EDX) at an accelerating voltage of 15 kV. Dot mapping analysis was also conducted for evaluating the elemental dispersion of hydrogel structure. To evaluate pore size distribution, the cross-section area of samples was assessed using BEL View image analysis software (6.2.2.1). Assuming a sphere-like pore shape, the equivalent diameter was reported as the pore size. The distribution of pore sizes in the hydrogel samples was supposed to be Gaussian and the following equations were used to determine the pore size polydispersity index (*PSPI*):

1 $$PSPI=\frac{{\sum {m}_{i}{D}_{i}^{2} \left/ \sum {m}_{i}\right.}}{{\left(\sum {m}_{i}{D}_{i} \left/ \sum {m}_{i}\right.\right)}^{2}}$$

where “n” and “d” are the number and diameter of pores, respectively. PSPI value equals 1 on the condition that all pores in a sample with symmetric sizes. In addition, micro-CT scanning (LOTUS-NDT, Behin Negareh Co., Iran) was used to evaluate morphology as well as porosity assessment of scaffolds as described in detail in our previously published research [83].

### FT-IR

FT-IR is an analytical technique used to identify the functional groups present in organic and inorganic compounds and investigate intermolecular interaction between various components. Infrared spectra were recorded with an FT-IR spectrophotometer (TENSOR 27, Germany) in the range of 500-4000 cm^-1^ with 4 cm^-1^ resolution and 24 scans.

### In vitro swelling evaluation and gel content

To assess fluid absorbed ability, the swelling evaluation was performed in PBS at the physiological conditions. Cylindrical hydrogels of 1 cm in diameter and 10 mm in height were prepared by pouring the mixture of each sample into molds, adding a 0.2 M crosslinking agent, and incubating at 37 °C for 2 h to complete the gelation process. Then, gelled hydrogels were weighed and the initial weight was recorded as *W*_*I*_. Samples were immersed in PBS solution at 37 °C for different time intervals of 24, 48, 72, and 96 h. Each assay was performed in quadruplicate. After the completion of the incubation period, samples were taken out from the buffer solution, the surface was lightly cleared of water by blotting paper and weighted (*W*_*F*_). All measurements were carried out three times individually. The swelling ratio was calculated from the following formula:

2 $$Swelling{\mkern 1mu} \,ratio\left( \% \right) = \left( {\frac{{W_{{\text{F}}} - W_{{\text{I}}} }}{{W_{I} }}} \right) \times 100$$

Gel fraction refers to the crosslinking degree formed in the polymeric structure of hydrogel. The amount of fractions indicates the number of crosslinks formed. The freeze-dried hydrogels were immersed in deionized water at room temperature for 48 h. The samples were subsequently dried in a vacuum oven at 70 °C for 24 h to achieve constant weight values. The gel fraction was estimated as follows:

3 $$\begin{aligned}{Gel\,fraction \,{(\%)}}=\frac{Wd}{{W}_{s}}\times 100\end{aligned}$$

where *W*_*s*_ refers to the initial weight of the dried sample and *W*_*d*_ is the weight of the dried insoluble part of the sample after water extraction.

### Mechanical property of hydrogels

To evaluate the compressive strain of hydrogel samples, the polymer solution was gelled in PDMS cylinder molds (diameter, 10 mm; height, 1 cm) by the addition of crosslinking agents. The mixtures were placed at 37 °C for 2 h to complete the gelation process, followed by incubation at standard condition (at 37 °C with 5% CO_2_) overnight. The gelled samples were blotted lightly with KimWip and tested at a rate of 2.0 mm min^−1^ using a material testing machine (Zwick/roell Z010, Germany).

### In vitro degradation rate assessment

Polymer solutions were polymerized as mentioned swelling evaluation section. A known quantity of gelled hydrogels (W_i_ = 1 g) were incubated in PBS buffer containing 1 mg ml^−1^ lysozyme. At weekly intervals, to assess the degradation rate, the samples were taken from buffer solution, completely rinsed, lyophilized for 48 h, and weighted. The degradation rate was calculated corresponding to the average data of three specimens of each hydrogel scaffold as Eq. (4).

4 $$Degradation\,rate (\%)={\left(\frac{{W}_{o}-{W}_{d}}{{W}_{o}}\right)} \times 100$$

where *W*_*d*_ and *W*_*o*_ are the weights in each sample at each time interval and initial weight, respectively.

### Cell culture and cell-laden microcapsules construction

In this study, osteoblast-like MG-63 cells were cultured in DMEM/LG supplemented with 10% (v/v) FBS and 1% (v/v) Pen-Strep solution and incubated at 37 °C under a 95% relative humidity and 5% CO_2_. The medium was refreshed every three days. MG-63 cells were subjected to sub-culture at 70–80% of confluence. For this purpose, the cells were detached using a 0.25% Trypsin–EDTA solution. The cell-laden microcapsules were fabricated by electrostatic encapsulation technique using a high voltage power supply (Vita Teb, Iran). 2 × 10^6^ cells mL^−1^ were suspended in Alg-nHA-Col solution. The mixture of cells and polymer solution was then dropped slowly through a 25 G needle into a gelling bath containing 0.2 M calcium chloride (CaCl_2_), barium chloride (BaCl_2_), and strontium chloride (SrCl_2_) in CF-KRH solution under sterile conditions. In this study, the extrusion flow rate and voltage were adjusted to 0.2 mL min^−1^ and 8 kV, respectively. The distance of the needle from the gelling bath was 5 cm to fabricate Alg-nHA-Col spheres. At the end of the encapsulation process, microcapsules were rinsed with CF-KRH buffer solution and culture medium to remove the residual cross-linking agent. Afterward, the microcapsules were suspended in low-glucose DMEM medium culture. The mean size of microcapsules generated was 400 ± 50 µm with an average of 50 microcapsules. Additional file Figure S1 illustrates the schematic of ionic gelation of alginate-based composite in the presence of Ca^2+^_,_ Ba^2+^, and Sr^2+^ divalent ions.

### In vitro cytocompatibility evaluation of cell-seeded microcapsules

The metabolic activity and proliferation of cells inside hydrogels were assessed using an MTT assay. At respective time points, the medium was removed from samples, the number of microspheres counted, washed twice with CF-KRH buffer solution, and subsequently treated with 1 mL of 3-(4,5-dimethylthiazol-2-yl)-2,5-diphenyltetrazolium bromide (MTT, 5 mg mL^−1^ in medium) at 37 °C for 24 h. Thereafter, samples were maintained in a fully humidified atmosphere of 5% CO_2_. Afterward, the samples were centrifuged at 3000 rpm for 6 min. The MTT solution was discarded and replaced with 1 mL DMSO, following incubation for 15 min in an incubator. Upon dissolving formazan crystals, the absorbance of samples (optical density) was eventually measured on UV/Vis spectrophotometer (CE2501, Japan) at 570 nm. OD values were normalized to the number of microcapsules per sample.

### Viability assay

The viability of encapsulated cells was assayed by a live cell staining. Microcapsules were taken from the medium, rinsed with PBS, and resuspended in 1 mL working solution supplemented with Calcein-AM (CAM; 1:1000) at 37 °C for 30 min. DAPI staining according to the manufacturer’s instruction was conducted to identify the cell nucleus in live cells. The stained samples were visualized by fluorescence microscopy (Model: BZ-9000; KEYENCE; Japan).

### Measuring oxidative stress in encapsulated cells

The activity of antioxidant enzymes such as SOD, GPx, and TAC was detected according to the previously published data [84]. The values were expressed as IU per mg of total protein.

### ALP activity determination

ALP activity was assessed with BCIP/NBT alkaline phosphatase color development kit. Briefly, at pre-determined times, the samples were rinsed with CF-KRH buffer solution, fixed in 4% formaldehyde solution for 2 min, followed by suspending in 0.2% Triton X-100 for 10 min to cells permeability. Then, BCIP/NBT substrate solution was added to samples, followed by 15 min incubation at 37 °C in a dark place. Images were captured on a phase-contrast microscope (OLYMPUS IX71). ALP activity in samples was assayed regularly by detecting the release of *p*-nitrophenol from *p*-nitrophenylphosphate. To quantification of ALP activity, the samples were rinsed twice with CF-KRH buffer solution, fixed with formaldehyde (4%) for 2 min, and followed by incubating at 37 °C for 1 h in a lysis buffer (1% Triton X-100, 8 mM MgCl_2_.6H_2_O, 150 mM NaCl, 50 mM Tris-based, pH 10) with gently shake. Thereafter, samples were resuspended in 500 µL *p*-NPP substrate solution and incubated for 30 min under standard culture conditions in the dark. 500 µL of an ice-cooled NaOH (0.1 M) was added to terminate the enzymatic activity. Absorbance at 405 nm was measured on the UV/Visible spectrophotometer. The absorbance values were expressed per the number of sample microcapsules.

### Alizarin red S staining and mineral characterization

The existence of mineral formation was investigated in hydrogels on days 7, 14, 21, and 28. At each time point, the medium was discarded and after rinsing samples with CF-KRH buffer solution, microspheres were fixed by 4% formaldehyde solution (at room temperature for 15 min). Thereafter, the samples were stained with 40 mM Alizarin red S solution (pH 4.1–4.3) and incubated in the dark for 20–30 min at 37 °C. After that, the staining solution was discarded and samples were washed with deionized water and observed by a phase-contrast microscope (OLYMPUS IX71). Cell-free microcapsules were subjected to a similar procedure of staining. In this experiment, cell-free microcapsules were negative for Alizarin red S staining.

### Hematoxylin–Eosin and von Kossa staining

Sections at 5 μm thickness were prepared from 21-day-old microspheres cross-linked with Ca^2+^_,_ Ba^2+^, and Sr^2+^ ions to assess cell distribution and calcium deposition using Hematoxylin–Eosin and von Kossa staining, respectively according to previously published data [83].

### PCR array analysis of Wnt signaling

The possible impact of three different hydrogels on Wnt signaling transduction pathways was assessed using PCR array analysis. Twenty one-day after the culture of MG-63 cells inside Alg-nHA-Col hydrogels cross-linked with Ca^2+^, Ba^2+^, and Sr^2+^, cells were decapsulated and RNA content extracted using Qiagen RNAeasy kit. Using RT^2^ First Strand Kit (SABiosciences), the expression of Wnt signaling genes was investigated using the Human Wnt RT^2^ Profiler PCR Arrays (PAHS-043Z, SABiosciences). Real-time PCR reaction was performed on the Light Cycler 480 System II (Roche) and data were analyzed using 2^−ΔΔCT^ (Light Cycler 480 quantitative software) in comparison with control housekeeping genes. Web-based RT^2^-based PCR array analysis (SABiosciences) was used to represent the data as a fold change expression. Differences in expression more than twofold were accepted as the cut-off value. *p* < 0.05 was considered statistically significant. This assay was performed in triplicate.

### Western blot analysis

Western blotting was used to assess the osteo-chondrogenic activity in cells inside Alg-based microspheres after 21 days. Upon the completion of the incubation period, 10 µg of protein from each group was used. For electrophoresis, samples were loaded in 10% SDS–polyacrylamide gel and separated. The protein bands were transferred onto PVDF using 300 mA for 1 h. The procedure was continued with the blocking of PVDF in 5% non-fat dry milk and incubation with anti-human COL1A1, OCN, and SOX-9 antibodies (All antibodies were purchased from Sana Cruz Inc.). To exclude background staining, membranes were incubated in TBST three times (each for 10 min). Thereafter, membranes were incubated with appropriate secondary HRP-conjugated antibodies at room temperature for 1 h. Immunoreactive bands were visualized using an ECL detection kit. Semi-quantitative analysis of each band was done using ImageJ software (NIH) and data were normalized to β-actin.

### In vitro migration assay using IHC staining

To this end, we prepared a bone-like module. For this purpose, microspheres were developed using three different ionic cross-linkers as above-mentioned. In the next step, developed microspheres were covered by a cylindrical Alg-nHA-Col matrix (5 × 10 mm) with similar ionic cross-linkers. The final size of modules reached 5 × 10 mm. After being incubated at 37˚C for 21 days, the modules were stained using anti-MMP-2 (sc-53630), and -9 (Cat No: sc-21733)] antibodies purchased from Santa Cruz Biotechnology Inc. After that, 5 µm-thick slides were exposed to 3% H_2_O_2_ for 20 min and 1% bovine serum albumin (Sigma-Aldrich). The procedure was followed by the addition of primary antibodies according to the manufacturer’s recommendation. After several PBS washes, the slides were stained by an HRP-conjugated antibody and visualized under microscopy.

### In vivo osteogenesis assay in a rat model

All procedures related to animal studies were approved by the Local Research Committee of Sahand University of Technology and rats were treated under the published protocols of “The Care and Use of Laboratory Animals (NIH Publication No. 85–23, revised 1996)”. The animals were maintained in a standard condition with a temperature around 25 ± 2 °C and humidity of 50 ± 5%, and a 12 h light/12 h dark cycle. In this study, 12 male Wistar rats were randomly allocated into three groups (each in 4) to assess the osteogenesis capacity of microspheres using a critical-sized cranial bone defect. Cells were encapsulated in modular hydrogels with a 5 mm diameter critical size and cross-linked with SrCl_2_ according to the in vitro data. Before the surgical procedure, rats were anesthetized by injection of 50 mg kg^−1^ Ketamine hydrochloride (ChemiDaru, Iran) and 5 mg kg^−1^ Xylazine (ChemiDaru, Iran). Approximately 5 mm diameter critical size defects were created on a calvarial bone micro bone drilling machine (Escort-III, Seayang Microtech, Korea). Calvarial defects were filled with modular scaffolds including microcapsules without cells, and modular scaffolds including cell-laden microcapsules. In the control rats, the defects were not filled with any materials. All incisions were sutured using absorbable surgical sutures (3–0 Chromic Gut). Eight weeks after the operation, rats were euthanized using an overdose of Ketamine and Xylazine, and newly-reconstructed bone mass was analyzed by the radiographic scans using CBCT Newtom GIANO/VG3- (Quantitative Radiology, Imola, Italy) and micro-CT scanning (LOTUS-NDT, Behin Negareh Co., Iran). For histological evaluation, specimens were fixed in formalin solution (10%) and decalcified using EDTA (10%). Following the fixation in 10% formalin solution and dehydration in an ascending series of ethanol, paraffin-embedded blocks were prepared. 5 µm thick sections were stained with H&E solution. For immunohistochemical analysis (IHC), de-paraffinized sections were exposed to hydrogen peroxide (3%) for 10–15 min to neutralize endogenous peroxidase activity. After antigen retrieval, slides were incubated in a solution containing an anti-osteonectin antibody (dilution: 1:200; Santa Cruz Biotechnology) for 1 h. After three-time PBS washes, samples were incubated with secondary HRP-conjugated antibody for 1 h and washed with PBS. Here, we used 3, 3’-Diaminobenzidine as chromogenic substrate.

### Statistical analysis

Statistical analysis was performed by one-Way ANOVA with a Turkey post hoc test using GraphPad Prism (version 8.0.2; GraphPad Software Inc.). Mean values were calculated from three independent triplicates otherwise mentioned. Data are shown as mean ± SD. The differences among samples were considered statistically significant at p values below 0.05.

## Supplementary Information

**Additional file 1: Figure S1** Schematic representation of the cell microencapsulation process by different divalent cations as crosslinker agents.

**Additional file 2: Figure S2** Clustergram of Wnt signaling pathway. The color saturation reflects the magnitude of the change in gene expression. Green squares designate lower gene expression (ratios <2), black squares designate genes equally expressed (ratios near 2), red squares illustrate higher gene expression in the experimental samples (ratios >2), and gray squares indicate insufficient or missing data.

**Additional file 3: Table S1** Different Wnt signal transduction pathways.

## Data Availability

All data generated or analyzed during this study are included in this published article.

## Abbreviations

Alg-nHA-Col: Alginate, nano-hydroxyapatite, and collagen
Alg: Alginate
ALP: Alkaline phosphates
Col: Collagen type I
DMSO: Dimethyl sulfoxide
ECM: Extracellular matrix
CF-KRH: Calcium-free Krebs Ringer HEPES-buffered saline
FBS: Fetal Bovine Serum
FT-IR: Fourier transform infrared spectroscopy
Gel: Gelatin
GPx: Glutathione peroxidase
HA: Hydroxyapatite
IHC: Immunohistochemistry
DMEM/LG: Low glucose-content dulbecco’s modified eagle’s medium
MMP-2, and -9: Metalloproteinase-2 and -9
micro-CT: Micro-computed tomography
(nHA): Nano-hydroxyapatite
OCN: Osteocalcin
OCN: Osteocalcin
Pen/Strep: Penicillin/Streptomycin
PBS: Phosphate-Buffered Saline
*p*-NPP: *p*-Nitrophenylphosphate
PVDF: Polyvinylidene difluoride membrane
RIPA: Radioimmunoprecipitation assay buffer
SOD: Superoxide dismutase
3D: Three-dimensional
TAC: Total Antioxidant
TBST: Tris-buffered saline containing 0.1% Tween 20
Wnt: Wingless-related integration site
PCR: Polymerase chain reaction
