# Supplementary figures and images for: Interaction of alginate with nano-hydroxyapatite-collagen using strontium provides suitable osteogenic platform
Source: J Nanobiotechnology. 2022 Jun 28;20:310. doi: 10.1186/s12951-022-01511-9 (PMC9238039; doi:10.1186/s12951-022-01511-9)

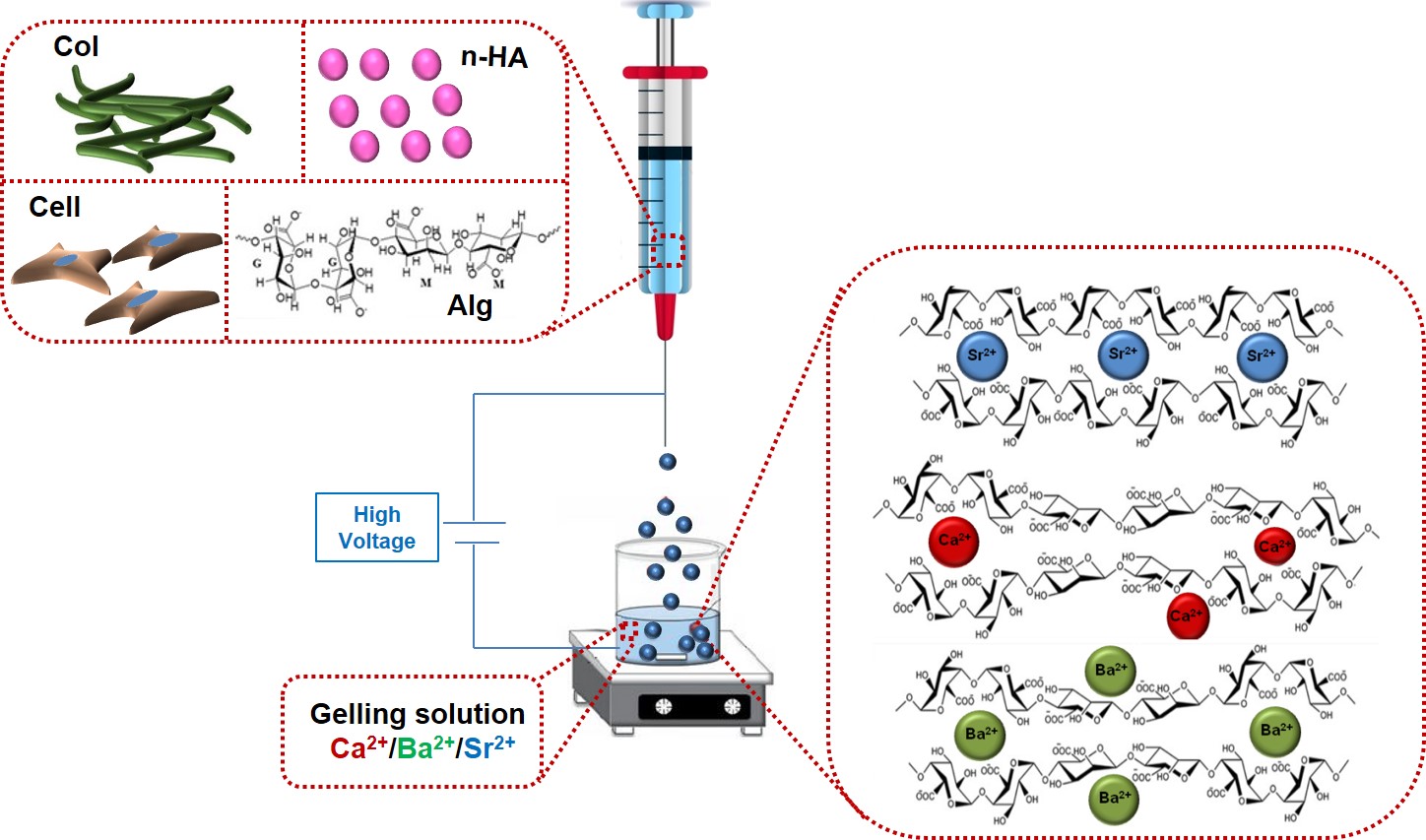

Supplement: Supplementary file 1 — Additional file 1: Figure S1 Schematic representation of the cell microencapsulation process by different divalent cations as crosslinker agents. [file 12951_2022_1511_MOESM1_ESM.jpg]

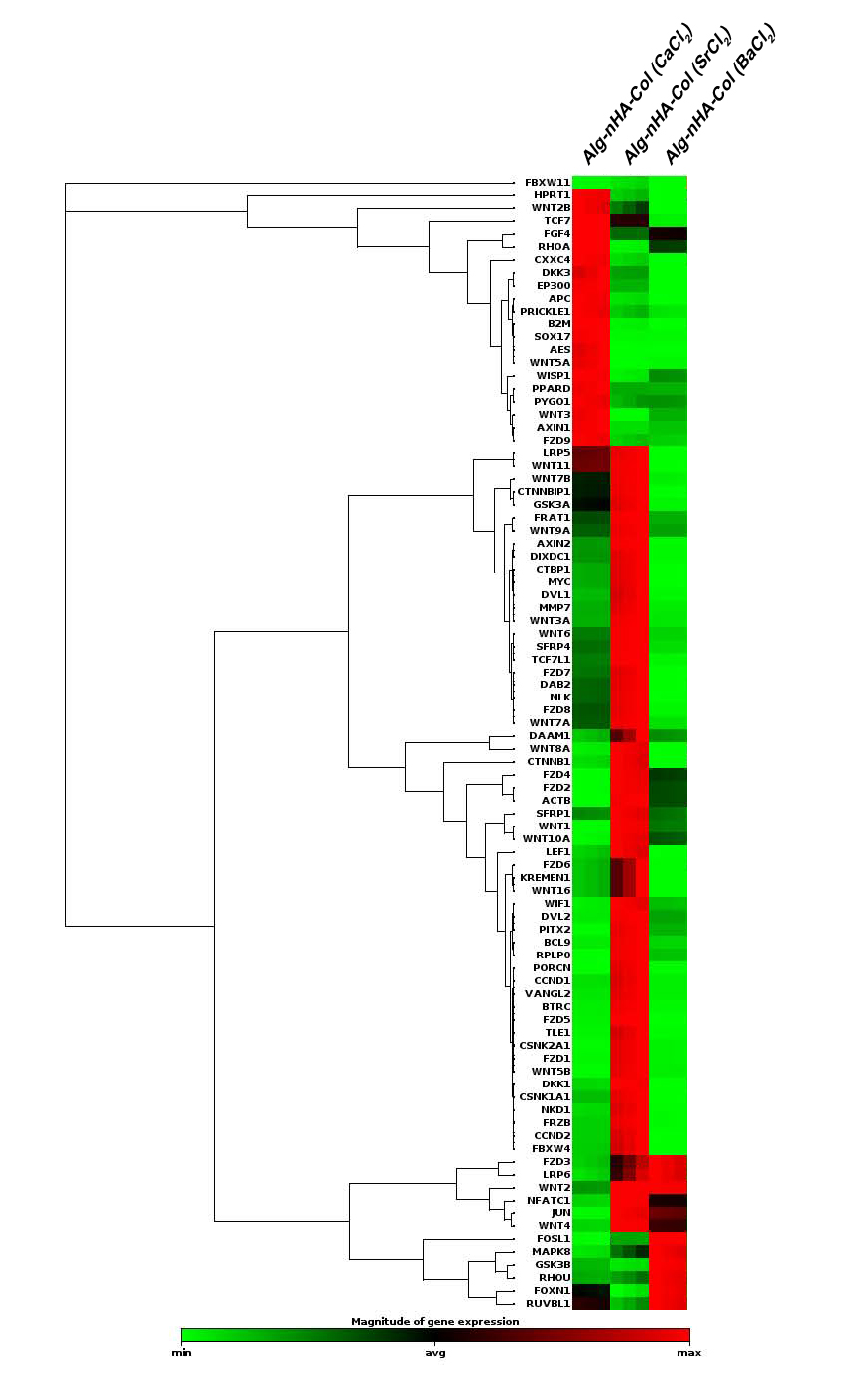

Supplement: Supplementary file 2 — Additional file 2: Figure S2 Clustergram of Wnt signaling pathway. The color saturation reflects the magnitude of the change in gene expression. Green squares designate lower gene expression (ratios <2), black squares designate genes equally expressed (ratios near 2), red squares illustrate higher gene expression in the experimental samples (ratios >2), and gray squares indicate insufficient or missing data. [file 12951_2022_1511_MOESM2_ESM.jpg]
