# Supplementary material for: Interaction of alginate with nano-hydroxyapatite-collagen using strontium provides suitable osteogenic platform
Source: J Nanobiotechnology. 2022 Jun 28;20:310. doi: 10.1186/s12951-022-01511-9 (PMC9238039; doi:10.1186/s12951-022-01511-9)
Supplement: Supplementary file 3 — Additional file 3: Table S1 Different Wnt signal transduction pathways. [file 12951_2022_1511_MOESM3_ESM.docx]

**Additional file Table S1**. Different Wnt signal transduction pathways

| **Different Wnt signal transduction pathways** | **List of genes** |
| --- | --- |
| Canonical WNT Signaling | APC, AXIN1, AXIN2, CSNK1A1, CTBP1, CTNNB1, CTNNBIP1 (ICAT), DKK1,DKK3, DVL1, DVL2, EP300, FRAT1, FZD1, FZD2, FZD3, FZD4, FZD5, FZD6, FZD7, FZD8, FZD9, GSK3B, LEF1, LRP5, LRP6, NKD1, PORCN, RUVBL1, SFRP1, SFRP4, SKP2, SOX17, TCF7, TCF7L1, WIF1, WNT1, WNT10A, WNT2, WNT2B, WNT3, WNT3A, WNT4, WNT6, WNT7A, WNT7B, and WNT8A |
| Planar Cell Polarity (PCP) | DAAM1, DVL1, DVL2, MAPK8 (JNK1), NKD1, PRICKLE1, RHOA, VANGL2, WNT9A.  WNT/Calcium Signaling FZD2, NFATC1, WNT1, WNT10A, WNT11,WNT2, WNT2B, WNT3, WNT3A, WNT4, WNT5A, WNT5B, WNT6, WNT7A, WNT7B, WNT8A, and WNT9A |
| WNT Signaling Negative Regulation | APC, AXIN1, AXIN2, BTRC (bTrCP), CCND1, CTBP1, CTNNBIP1 (ICAT), DKK1, DKK3, FBXW11, FRZB (FRP-3), KREMEN1, LRP6, NKD1, NLK,SFRP1, SFRP4, SOX17, and WIF1 |
| WNT Signaling Target Genes | AXIN2, BTRC (bTrCP), CCN4, CCND1, CCND2, DAB2, FOSL1 (FRA-1), JUN, MMP7, MYC, PITX2, and PPARD |
| Cell Fate | CTNNB1, DKK1, WNT1, WNT3, and WNT3A |
| Tissue Polarity | AXIN2, FZD2, FZD3, FZD5, FZD6, and VANGL2 |
| Cell Growth & Proliferation | APC, CCN4, CCND1, CCND2, CTBP1, CTNNB1, CTNNBIP1 (ICAT), DAB2, EP300, FGF4, FOSL1 (FRA-1), FZD3, JUN, LRP5, MMP7, MYC, PPARD, and WNT3A |
| Cell Migration | APC, DKK1, LRP5, LRP6, RHOA, and WNT1 |
| Cell Cycle | APC, BTRC (bTrCP), CCND1, CCND2, CTNNB1, EP300, FOSL1 (FRA-1), JUN, MYC, RHOA, RUVBL1, and TCF7L1 |
| Cellular Homeostasis | APC, FZD2, JUN, and MYC |
| Pathway Activity Signature Genes | BOD1, CALM1, CCND1, CCND2, CHSY1, CXADR, CYP4V2, HSPA12A, LEF1, MT1A, MTFP1, MTSS1, MYC, NAV2, PRMT6, and SKP2 |
